# Expression of the *Aspergillus terreus* itaconic acid biosynthesis cluster in *Aspergillus niger*

**DOI:** 10.1186/1475-2859-13-11

**Published:** 2014-01-17

**Authors:** Laura van der Straat, Marloes Vernooij, Marieke Lammers, Willy van den Berg, Tom Schonewille, Jan Cordewener, Ingrid van der Meer, Andries Koops, Leo H de Graaff

**Affiliations:** 1 Microbial Systems Biology, Laboratory of Systems and Synthetic Biology, Wageningen University, Dreijenplein 10, Wageningen 6703 HB, Netherlands; 2 Bioscience, Plant Research International, Droevendaalsesteeg 1, Wageningen 6708 PB, The Netherlands

**Keywords:** *Aspergillus niger*, *Aspergillus terreus*, *cis*-aconitate decarboxylase *cadA*, Mitochondrial transporter *mttA*, Plasma membrane transporter *mfsA*, Itaconic acid

## Abstract

**Background:**

*Aspergillus terreus* is a natural producer of itaconic acid and is currently used to produce itaconic acid on an industrial scale. The metabolic process for itaconic acid biosynthesis is very similar to the production of citric acid in *Aspergillus niger.* However, a key enzyme in *A. niger*, *cis*-aconitate decarboxylase, is missing. The introduction of the *A. terreus cadA* gene in *A. niger* exploits the high level of citric acid production (over 200 g per liter) and theoretically can lead to production levels of over 135 g per liter of itaconic acid in *A. niger*. Given the potential for higher production levels in *A. niger*, production of itaconic acid in this host was investigated.

**Results:**

Expression of *Aspergillus terreus cis-*aconitate decarboxylase in *Aspergillus niger* resulted in the production of a low concentration (0.05 g/L) of itaconic acid. Overexpression of codon-optimized genes for *cis-*aconitate decarboxylase, a mitochondrial transporter and a plasma membrane transporter in an oxaloacetate hydrolase and glucose oxidase deficient *A. niger* strain led to highly increased yields and itaconic acid production titers. At these higher production titers, the effect of the mitochondrial and plasma membrane transporters was much more pronounced, with levels being 5–8 times higher than previously described.

**Conclusions:**

Itaconic acid can be produced in *A. niger* by the introduction of the *A. terreus cis-*aconitate decarboxylase encoding *cadA* gene. This results in a low itaconic acid production level, which can be increased by codon-optimization of the *cadA* gene for *A. niger.* A second crucial requirement for efficient production of itaconic acid is the expression of the *A. terreus mttA* gene, encoding a putative mitochondrial transporter. Expression of this transporter results in a twenty-fold increase in the secretion of itaconic acid. Expression of the *A. terreus* itaconic acid cluster consisting of the *cadA* gene, the *mttA* gene and the *mfsA* gene results in *A. niger* strains that produce over twenty five-fold higher levels of itaconic acid and show a twenty-fold increase in yield compared to a strain expressing only CadA.

## Background

Increased awareness of the environmental pressure caused by petroleum-based production processes and products has stimulated and intensified research on bio-based production methods and products. Efficient bio-based production is economically problematic due to the relative low-cost of petroleum-based chemicals and is also technically complex. The design and construction of efficient cell factories requires a modification of the host cell or chassis at a systems level rather than at a single gene level.

Itaconic acid or methylsuccinic acid is a C5 dicarboxylic acid. The methylene group of itaconic acid can participate in polymerization reactions. On the basis of this characteristic, itaconic acid can be used for the production of synthetic polymers [1]. Furthermore, it can used as a bioactive component in agriculture and pharmacy, as a medicine [2] and as a starting compound in enzymatic conversions to form useful poly-functional building blocks [3]. For all of these reasons, itaconic acid has been designated by the U.S. Department of Energy as one of the top twelve building-block chemicals that can be produced from plant biomass sugars *via* a fermentative process [4].

**Figure 1:**
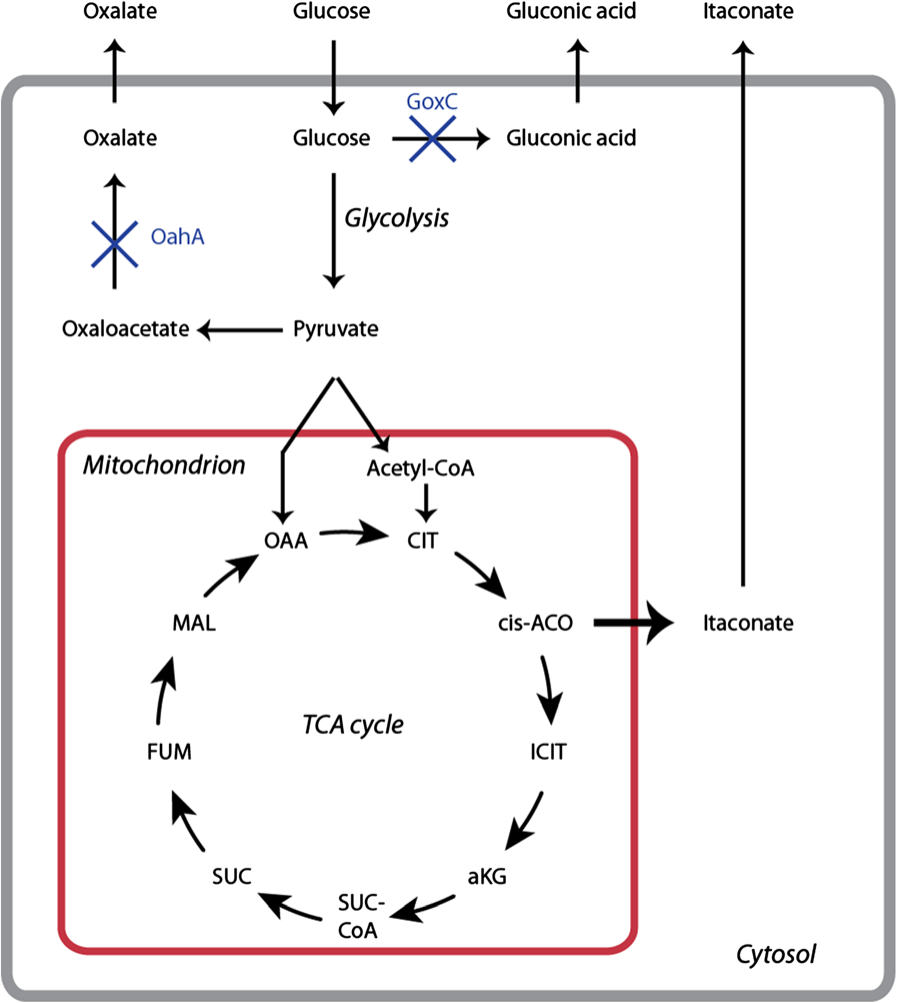
**Metabolic pathway for itaconic acid production via the different compartments in the specific *A. niger *** **strain.**

Currently, *A. terreus* is used for the commercial production of itaconic acid by submerged fermentation [2,5]. The pathway for the production of itaconic acid is a metabolic variant of the pathway for citric acid production in *A. niger* (Figure 1). Citric acid is produced commercially using *A. niger*, reaching production levels over 200 g/L [6], which, in a molar ratio, corresponds to over 135 g/L itaconic acid. *A. terreus* reaching itaconic acid titers of 80 g/L shows the potential of *A. niger* to reach far higher production titers. A second advantage is that the existing citric acid fermentation infrastructure can be used for this *A. niger*-based fungal itaconic acid cell factory.

*A. niger* does not naturally produce itaconic acid because it lacks the essential enzyme *cis*-aconitate decarboxylase. The *cadA* gene encoding this enzyme in *A. terreus* has been identified using different approaches, including an enzyme purification approach [7] and a clone-based transcriptomics approach [8]*.* The expression of the *cadA* gene in *A. niger* leads to extremely low levels of itaconic acid production (0.05 g/L), indicating that the sole expression of the enzyme is insufficient for efficient production of itaconic acid. In the *A. terreus* genome, the *cadA* gene is located close to the lovastatin cluster [9] and flanked by a putative mitochondrial transporter (*mttA)* and a putative plasma membrane transporter (*mfsA)*. The co-regulation of these transporters with *cadA*, as reported by Li et al. [8], suggested that the putative mitochondrial transporter might be involved in itaconic acid production in *A. terreus*. Recently, Li et al. [10] showed that the effect of these putative transporters on itaconic acid production in *A. niger* resulted in a slight increase in itaconic acid production levels. However, the maximum titer of 1.5 g/L itaconic acid that was reached is far from the theoretical titer of over 135 g/L under conditions of high citric acid production.

For our studies, we have used a specific mutant of *A. niger* to serve as a chassis for the production of itaconic acid. This strain carries two specific mutations, one in the *oahA* gene encoding oxaloacetate hydrolase and one in the *goxC* gene encoding glucose oxidase. This strain has certain advantages; the production of by-products is reduced because it is not able to produce oxalic acid or gluconic acid. As a result, this leaves more carbon available for citrate and itaconate production. Finally, as reported by Ruijter et al., strains carrying both the *oahA* mutation and the *goxC* mutation are insensitive to Mn^2+^ ions in the medium, which results in constitutive citrate production irrespective of the fermentation regime [11]. In this study, we use a robust fermentation regime developed by van der Veen et al. [12] that is optimized to reduce variance in the experiments. In the fermentation medium, sorbitol is used as the main carbon source as it is essentially non-inducing and non-repressing for the D-xylose inducible *xlnD* promoter [13]. Sorbitol is metabolized to form fructose [14], which is phosphorylated by hexokinase to fructose-6-phosphate and further metabolized *via* the glycolysis pathway and the TCA cycle. In the TCA cycle, citrate is converted into isocitrate in a reaction that yields *cis*-aconitate as an intermediate. Itaconic acid can be formed from *cis*-aconitate by a *cis*-aconitate decarboxylase-catalyzed reaction.

In our study, we show that the overexpression of the codon-optimized *cadA*, *mttA* and *mfsA* genes in the oxaloacetate hydrolase- and glucose oxidase-deficient strain leads to increased yields and itaconic acid production titers. At these higher production titers, the effect of the mitochondrial and plasma membrane transporters is much more pronounced than previously described [10].

## Results and discussion

Our strategy for the design of a fungal cell factory was based on the use of a specific chassis for the production of itaconic acid in *A. niger*. The *A. niger* strain that we chose is a mutant strain that is not able to produce oxalic acid or gluconic acid due to mutations in the *oahA* and *goxC* genes, respectively. This is an important advantage because this strain does not produce these unwanted side products. Due to the reduced formation of by-products, more carbon can be converted into the final product - itaconic acid. The *oahA* mutation also leads to constitutive citric acid production that is insensitive to the presence of metal ions, as discovered by Ruijter et al. [11]. The constitutive production of citric acid is a great benefit because itaconic acid production is directly derived from citric acid production.

### Expression of the *A. terreus cadA* gene in *A. niger*

The gene encoding *cis-*aconitate decarboxylase was identified in the *A. terreus* genome using a proteomics approach in which the enzyme was partially purified. Both a cDNA fragment from *A. terreus* and a codon-optimized *cadA* synthetic gene were used for the expression of *cis-*aconitate decarboxylase in *A. niger* NW186. The *A. terreus* coding sequences have a slightly higher GC content in comparison to the *A. niger* coding sequences (56.2% vs 53.8%, respectively) [15]. This higher GC content is mostly found at the third position; in *A. terreus*, 65.3% GC and in *A. niger*, 59.3%. A total of 305 out of 490 codons were changed in the *cadA* sequence, including the codons to remove restriction enzyme sites.

**Figure 2:**
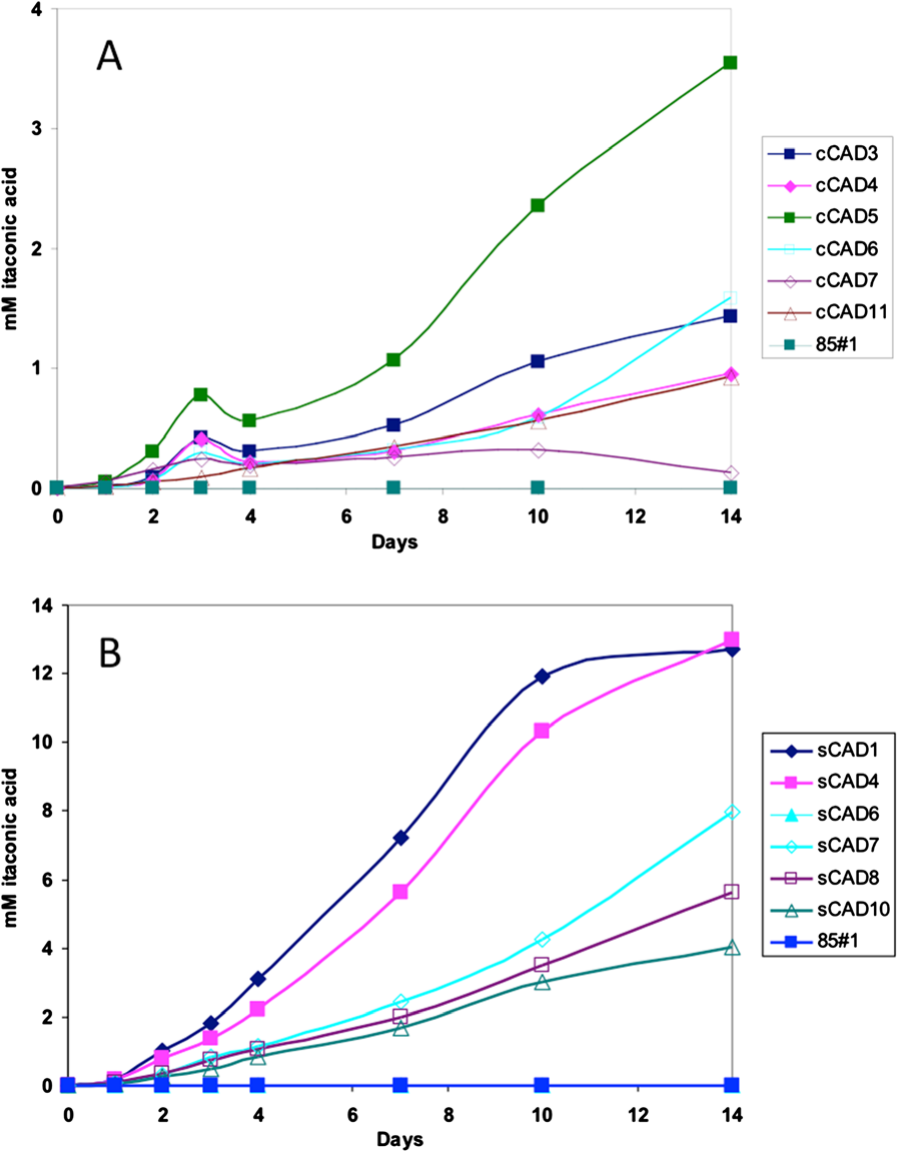
**Itaconic acid production in *A. niger *strains expressing *cis*-aconitate decarboxylase.** Production of itaconic acid (mM) in strains expressing *cadA* cDNA (cCAD) **(A)** and production of itaconic acid (mM) in strains expressing codon-optimized *cadA* synthetic DNA (sCAD) **(B)**.

The transformants from both plasmids yielded varying low amounts of itaconic acid. This variation in itaconic acid production could result from differences in copy numbers amongst the strains and variation in the site of integration of the construct in the different transformants. Surprisingly, codon-optimization of the *cadA* gene for *A. niger* resulted in a more than three-fold increase in itaconic acid production. The transformants containing the codon-optimized gene (sCAD) (Figure 2B) produced higher amounts of itaconic acid compared to the ones expressing the cDNA fragment (cCAD) (Figure 2A). Based on these results, the two putative transporters *mttA* and *mfsA* from *A. terreus* were also synthetically made and codon-optimized for expression in *A. niger*.

No itaconic acid production was detected in the *A. niger* strains that did not contain the *cis-*aconitate decarboxylase encoding gene. The sCAD4 strain was selected for our further studies.

### Copy number of the *cadA* gene in *A. niger* transformants

The copy number was determined by qPCR using genomic DNA as template. The Pfaffl method was applied to calculate the copy number [16]. The qPCR results for *cadA* were compared with those of the single copy gene *pkiA* in order to determine the copy number of *cadA*. For the transformants cCAD4, cCAD6 and cCAD11, expressing the *A. terreus cadA* cDNA, a copy number of 1 was determined. These strains also produced the same levels of itaconic acid for 10 days. After 14 days, the cCAD6 strain produced a higher level of itaconic acid. Of the strains expressing the *cadA* cDNA, cCAD3 and cCAD5 had the highest copy number at 4. These strains also produced the highest levels of itaconic acid of the strains that expressed the non-optimized cadA cDNA. The only atypical transformant was cCAD7, which had a copy number of 21 but produced hardly any itaconic acid. Although this was a striking result, it has previously been observed in *A. niger*[17].

The highest itaconic acid producing transformants, sCAD1 and sCAD4, expressed the synthetic codon-optimized *cadA* gene and had the highest copy numbers at 11 and 6, respectively. The transformants sCAD7, sCAD8 and sCAD10, which produced between 4 and 8 mM itaconic acid after 14 days, had copy numbers of 2, 2 and 4, respectively. Although the sCAD10 strain had 4 copies of *cadA*, it did not produce more than the strains with only 2 copies of *cadA*. In this particular case, the place of integration could negatively influence the level of expression compared to sCAD7 and sCAD8. The sCAD6 strain, which did not produce any itaconic acid, also did not have a copy of the *cadA* gene.

These results also suggest a positive effect of codon-optimization because the strains that produced the highest levels of itaconic acid, namely sCAD7, sCAD8 and sCAD10, were the strains expressing the codon-optimized *cadA* gene. The copy number of these strains was determined to be 2, 2 and 4, respectively. This is in contrast to the cCAD5 and cCAD3 strains, which carried 4 copies of the non-optimized *cadA* gene but produced less itaconic acid.

### Expression of the *A. terreus* itaconic acid biosynthesis cluster in *A. niger*

Based on our findings on the expression of the *cadA* in *A. niger*, we extended our studies by co-expressing the two putative transporter encoding genes flanking the *cadA* gene in the *A. terreus* genome*.* In these studies, we also used synthetic codon-optimized fragments of the *mttA* and *mfsA* genes for expression in the *A. niger* strain that contains the codon-optimized *cadA* gene, sCAD4. For our first analysis, these strains were grown in Erlenmeyer culture flasks to analyze the effects of the transporters that were introduced. In these experiments, strains that contained the *cadA* and *mfsA* genes produced slightly higher levels of itaconic acid compared to the sCAD4 strain carrying only the *cadA* gene (Table 1).

**Table 1:** Production of itaconic acid in Erlenmeyer cultures

	**Itaconic acid produced (mM)**	**Factor difference**
Control	0.13 ± 0.02	
*cadA* + *mfsA* 2.4	0.14 ± 0.01	1
*cadA* + *mfsA* 2.5	0.25 ± 0.06	2
*cadA* + *mttA* 1.1	0.98 ± 0.09	8
*cadA* + *mttA* 1.2	3.23 ± 0.94	25
*cadA* + *mttA* 1.4	0.74 ± 0.04	6
*cadA* + *mttA* 1.5	0.74 ± 0.00	6
*cadA* + *mttA* 1.6	1.21 ± 0.24	10

The itaconic acid production is given in mM in the CadA + MfsA and CadA + MttA transformants at 30 hours after induction. Measurements were carried out in duplicate; the ± represents the standard error of the mean.

The putative mitochondrial transporter *mttA* had a much more pronounced effect on itaconic acid production, as expression of this gene led to increased itaconic acid production in all transformants analyzed in comparison to the strain that had only the *cadA* gene. The increase found ranged between a factor of 6 and 25 (Table 1), which is far more than the previously described increase for an *A. niger* strain carrying only the *oahA* mutation and in which the genes were not codon-optimized [10].

We performed batch fermentations to study the improved itaconic acid production in a more controlled way. To investigate the effect of the *mttA* and *mfsA* transporters in the production of itaconic acid in *A. niger,* the best performing transformant of the *cadA* + *mttA* and *cadA* + *mfsA* strains were selected along with four transformants of newly constructed strains carrying the combination of *cadA*, *mttA* and *mfsA*. The parent strain sCAD4 was chosen as the control.

**Figure 3:**
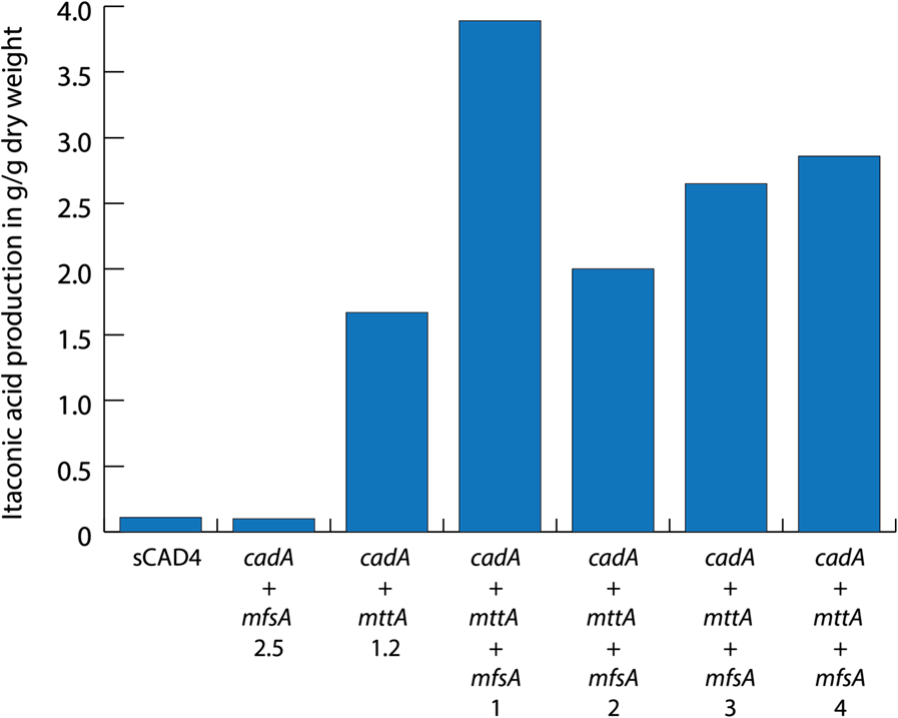
**Itaconic acid production in fermenter cultures.** The itaconic acid production is given in gram per gram dry weight at T = 78 hours after induction of the transformants carrying the complete itaconic acid biosynthesis cluster from *A. terreus* compared to the best performing CadA, CadA + MfsA and CadA + MttA transformants.

**Figure 4:**
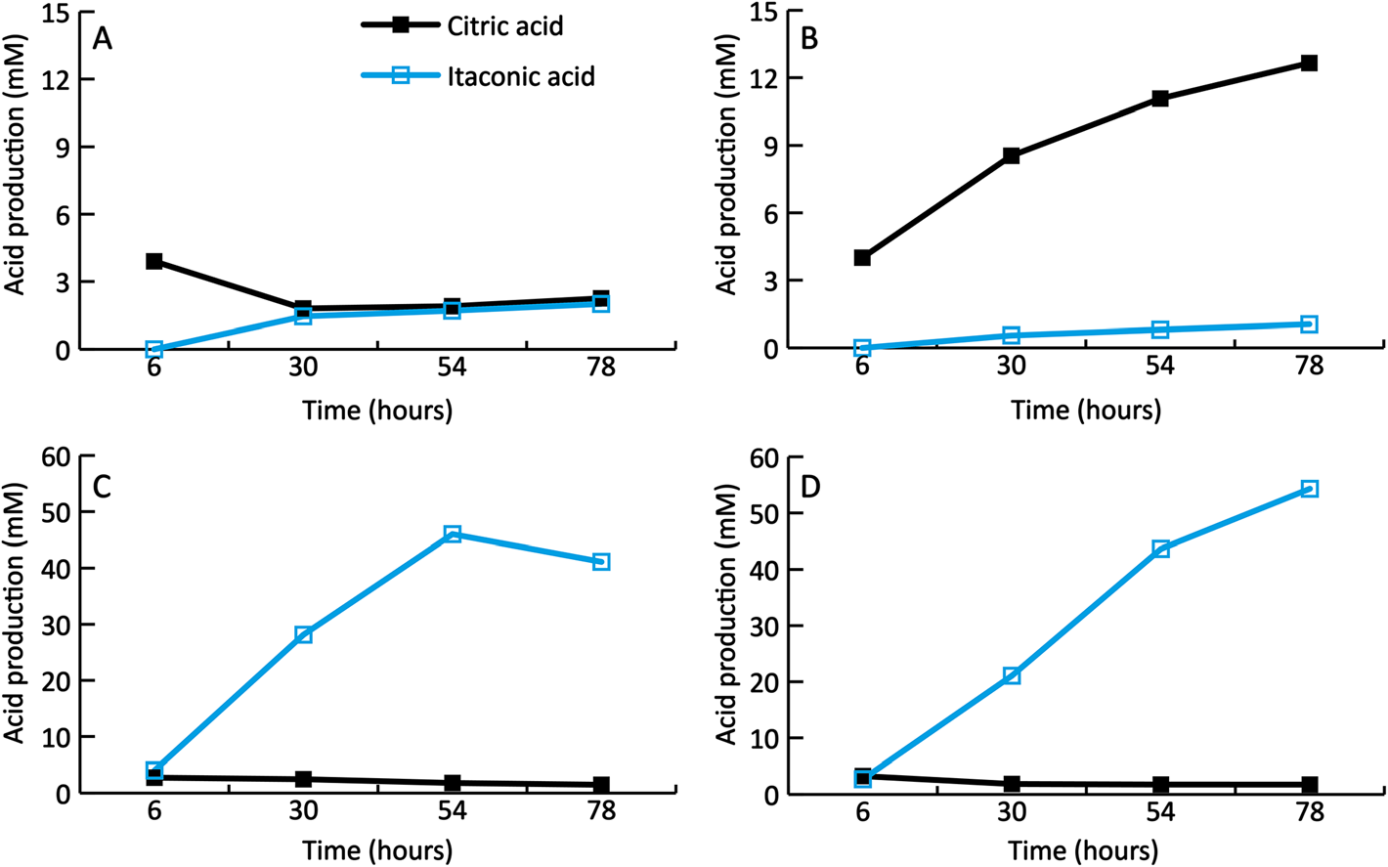
**Itaconic acid and citric acid production during fermentation of the different strains. A)** sCAD4 strain, **B) ***cadA* + *mfsA* 2.5, **C) ***cadA* + *mttA* 1.4 and **D) ***cadA + mttA + mfsA* 4. The black lines represent citric acid concentration and the blue line represents the itaconic acid concentration. The time is shown in hours (x-axis) and the concentrations are shown in mM (y-axis).

When grown in batch fermenters, we did not find the slight increase in itaconic acid production in the strains that co-expressed *mfsA* with *cadA* (Table 1, Figures 3 and 4.) that we found in the Erlenmeyer cultures. This was in contrast to previously published data in which nearly five-fold higher itaconic acid levels were found when *cadA* was co-expressed with *mfsA*[10]. However, we did find increased citric acid concentrations (Figure 4). This implies that this transporter is also able to export citric acid. Although we did not find a positive effect on the levels of itaconic acid production from strains expressing *mfsA* and *cadA*, we did find a positive effect on itaconic acid production levels in strains expressing *cadA*, *mttA* and *mfsA* in NW186 (Table 1, Figures 3 and 4). Apparently, the plasma membrane transporter MfsA is able to secrete both itaconic acid and citric acid. Obviously, *A. niger* is able to secrete itaconic acid without a heterologous plasma membrane transporter as is shown by the expression of *cadA* in *A. niger*. The transformant *cadA* + *mfsA* 2.5 did not show increased itaconic acid production in the fermenter studies, but because the itaconic acid levels are relatively low, it is possible that there was no bottleneck in itaconic acid export in this strain under these conditions.

In the case of the *cadA* + *mttA* + *mfsA* transformants, levels of itaconic acid production were strongly increased in comparison to the strain that only carried the *cadA* gene, with the highest increase being over 25-fold (Figure 3). These increased production levels were paralleled with an increased yield, defined as the product yield on the substrate in % (C-mol/C-mol), in the transformants that contained both putative transporters. The strain expressing only *cadA* gave a yield of approximately 1%, which is of the same order as was found by Li et al. [10], although they used different carbon sources and concentrations of carbon sources. The strongest increase in yield was caused by the expression of the mitochondrial transporter *mttA* in the sCAD4 strain, which resulted in a yield of 24%. But, when the complete itaconic acid cluster from *A. terreus* was expressed, the yield further increased to 32% in the best performing strain (Table 2).

**Table 2:** Comparison of itaconic acid producing strains

	**Itaconic acid production (g/L)**	**Sorbitol and xylose consumed (C-mmol)**	**Itaconic acid yield on consumed sorbitol and xylose (%)**	**Biomass (g dry weight per L culture broth)**	**Data from**
sCAD4	0.3	619	1.6	2.4	This research
*cadA* + *mfsA* 2.5	0.1	632	0.8	1.2	This research
*cadA* + *mttA* 1.2	5.4	843	24.4	3.2	This research
*cadA* + *mttA + mfsA* 1	5.6	806	26.8	1.6	This research
*cadA* + *mttA + mfsA* 2	6.0	848	27.4	3.1	This research
*cadA* + *mttA + mfsA* 3	5.5	702	30.0	2.1	This research
*cadA* + *mttA + mfsA* 4	7.1	844	32.1	2.6	This research
AB 1.13 CAD	0.9		1.0		Li *et al.*[13]
MTT 1.4	1.4		1.6		Li *et al.*[13]
MFS 3.9	1.4		1.6		Li *et al*. [13]
CAD + MTT + MFS_3	0.9		1.0		Li *et al.*[13]

The production of itaconic acid is given in g/L at 78 hours after induction. The yield in percentage (C-mol/C-mol) is calculated based on consumed sorbitol and D-xylose. All strains from Li et al. [13] are uridine prototrophs of AB 1.13.

Although several other acids including *cis*-aconitic acid, succinic acid, malic acid, pyruvic acid and α–ketoglutaric acid are secreted, itaconic acid was the predominantly produced acid in the highest producing strains. One exception was the strain expressing *cadA* in combination with *mfsA* where, instead of itaconic acid, citric acid was the predominantly produced acid (Table 3).

**Table 3:** Overview of extracellular acid concentrations (mM) measured in time during fermentation

		**Citric acid**	**Itaconic acid**	***cis*-aconitic acid**	**Succinic acid**	**Malic acid**	**Pyruvic acid**	**α-ketoglutaric acid**
6 h	sCAD4	3.9	ND	ND	ND	ND	ND	ND
	*cadA* + *mfsA* 2.5	4.0	ND	ND	ND	ND	ND	ND
	*cadA* + *mttA* 1.2	2.7	4.0	ND	0.2	ND	ND	ND
	*cadA* + *mttA + mfsA* 4	3.3	2.7	ND	ND	ND	ND	ND
30 h	sCAD4	1.8	1.45	0.3	ND	ND	2.4	ND
	*cadA* + *mfsA* 2.5	8.5	0.6	ND	ND	ND	ND	ND
	*cadA* + *mttA* 1.2	2.5	28.1	ND	0.7	ND	ND	1.5
	*cadA* + *mttA + mfsA* 4	1.9	21.1	1.2	ND	ND	0.8	ND
54 h	sCAD4	1.9	1.7	0.3	ND	ND	2.1	ND
	*cadA* + *mfsA* 2.5	11.1	0.8	ND	0.8	0.7	ND	ND
	*cadA* + *mttA* 1.2	1.8	46.0	ND	1.4	ND	ND	0.4
	*cadA* + *mttA + mfsA* 4	1.8	43.6	1.8	ND	ND	0.6	ND
78 h	sCAD4	2.3	2.0	0.3	ND	ND	2.2	ND
	*cadA* + *mfsA* 2.5	12.7	1.0	ND	0.9	0.7	ND	ND
	*cadA* + *mttA* 1.2	1.5	41.1	ND	0.9	ND	ND	ND
	*cadA* + *mttA + mfsA* 4	1.8	54.3	2.2	0.9	ND	ND	ND

ND: the compound was not detected in the sample.

The itaconic acid production levels obtained were relatively low compared to those in industrial production processes. It is not surprising that our strain was less efficient in itaconic acid production than the commercial *A. terreus* strain since the *A. niger* strain used is not producing high levels of citrate. The citrate concentrations produced by our strain are far lower than those obtained in an industrial environment using an industrial *A. niger* strain. Although itaconic acid production in *A. niger* was still far less efficient than in *A. terreus*, major improvements were made. Under lab conditions, we were able to improve the titer of itaconic acid produced in *A. niger* by a factor of over twenty-five and the yield by approximately twenty-fold. Compared to the values for itaconic acid production in *A. niger* that have been published [10], the strains we constructed showed a five-fold higher production level and a twenty-fold increase in yield. These improvements mainly resulted from the use of codon-optimized genes and from the increased efficiency of substrate-use by the elimination of oxalate and gluconate production.

## Conclusions

Itaconic acid can be produced in *A. niger* by the introduction of the *A. terreus cis-*aconitate decarboxylase encoding *cadA* gene. However, this results in very low production levels. The production levels can be increased if the *A. terreus cadA* gene is codon-optimized for *A. niger*.

When the expression of *cadA* in *A. niger* was combined with the expression of the *A. terreus mfsA* gene encoding a putative plasma membrane transporter, no effect on the production levels of itaconic acid was found. This suggests that the itaconic acid produced in *A. niger* is efficiently secreted by an endogenous *A. niger* transporter*.* The expression of *mfsA* in combination with *cadA* led to increased citrate production suggesting that MfsA is a transporter that is able to secrete citric acid as well as itaconic acid.

Our results show that in addition to the *cadA* gene, the *mttA* gene from *A. terreus* is also crucial for efficient itaconic acid production in *A. niger.* Expression of the *mttA* gene, encoding a putative mitochondrial transporter, in the strain that expresses CadA resulted in an over twenty-fold increased secretion of itaconic acid. Expression of the *A. terreus* itaconic acid cluster, consisting of the *cadA*, *mttA* and *mfsA* genes, led to *A. niger* strains with over twenty five-fold higher levels of itaconic acid and a 20-fold increase in yield when compared to a strain that expressed only CadA.

## Methods

### Strains and spore preparations

The fungal strains used in this study were *Aspergillus terreus* NRRL 1960 (Centraal Bureau voor Schimmelcultures, Baarn, The Netherlands, CBS 116.46) and *Aspergillus niger* NW186 (*cspA1*, *goxC17*, *prtF28* Δ*argB*, *pyrA6*), which is a *pyrA* mutant of *Aspergillus niger* NW185 [11].

To obtain spores, 20 spores per mm^2^ were plated onto complete medium plates [18], incubated for 5 days at 30°C and allowed to mature at 4°C for 24 h. The spores were harvested in 0.9% (w/v) NaCl and 0.005% (v/v) Tween-80, washed with 0.9% (w/v) NaCl and stored at 4°C until use.

### Fermentation and induction of itaconic acid production in *A. terreus* NRRL 1960

*A. terreus* was grown at 30°C and 200 rpm by inoculating spores (10^6^ spores per mL) in 100 mL pre-cultures in 1 L flasks containing 25 g/L glucose, 4.5 g/L MgSO_4_.7H_2_0, 0.4 g/L NaCl, 4 mg/L ZnSO_4_.7H_2_O, 100 mg/L KH_2_PO_4_, 2 g/L NH_4_NO_3_ and 0.5 g/L Corn Steep Liquor (CSL). After two days, a 10% (w/v) inoculum was transferred to the CAD production medium, as described by Cros and Schneider [19] with the following changes: 3 g/L NH_4_NO_3_ instead of urea and 1.5 g/L MgSO_4_.7H_2_O with a final pH of 2.0.

### Gene cloning and plasmid design

Standard methods were used to carry out DNA manipulations and *E. coli* transformations [20]. The gene encoding CadA was obtained by PCR from the *A. terreus* genome and cloned in a pUC19 derived vector under the control of the *A. niger pkiA* promoter [17] and the terminator of the *trpC* gene of *A. niger*. The codon-optimized *cadA* gene was synthesized by Geneart (Invitrogen, Carlsbad, CA, US) and cloned in pUC19. Codon-optimized genes *mttA* and *mfsA* were synthesized by DNA 2.0 (Menlo Park, USA) and cloned in a pUC19 derived vector under the control of a modified *xlnD* promoter and the terminator of the *xlnD* gene of *A. niger*[13]. The promoter modification involved the inactivation of CreA sites leaving the promoter inducible by D-xylose. For the construction of plasmid pLS001 the [p_xlnD* – MTT – t_xlnD] fragment was obtained by PCR using pMTT as a template and the following primers: LS_p_xlnD_HindIII_for (5′-GAG-AAA-GCT-TCG-AAT-GAG-GAG-GTG-TTG-CAG 3′) and LS_t_xlnD_XbaI_rev (5′-GAG-ATC-TAG-ACT-GCA-GTC-GCA-CTC-CCG-ACC 3′). This fragment was cloned into pMFS and digested with *HindIII* and *XbaI*.

The plasmids were propagated in DH5α *E. coli*, in LB medium (10 g/L Bacto tryptone, 5 g/L Yeast extract, 10 g/L NaCl) supplemented with the appropriate antibiotics (100 mg/L ampicillin, 50 mg/L kanamycin).

### Fungal transformation

For transformation of *A. niger,* protoplasts were generated using Novozyme 234. The *cadA*, *mttA* and *mfsA* genes were introduced in *A. niger* NW186 by co-transformation as previously described [21] using the pGW635 plasmid, which contains the *pyrA* gene [22] as a primary selection marker. The pLS001 plasmid was introduced in the *A. niger* strain containing the *cadA* gene by co-transformation using the pAL69 plasmid, which contains the a*rgB* gene as a selection marker. Selective MMS plates (6.0 g/L NaNO_3_, 1.5 g/L KH_2_PO_4_, 0.5 g/L KCl, 0.5 g/L MgSO_4_.7 H_2_O, 1 mL/L Vishniac, 325.2 g/L sucrose, 1.2% (w/v) agar, pH 6.0) were used to select for protoplasts that did take up the selection marker plasmid and possibly the plasmid of interest. Randomly, 20 colonies were picked from the transformation plates and replated on complete medium [18].

### DNA extraction and PCR of *cadA* + *mttA* and *cadA* + *mfsA* from *A. niger* transformants

DNA was extracted by adding 100 μL extraction buffer (100 mM Tris pH 8.0, 50 mM EDTA, 500 mM NaCl, 0.07% β-mercaptoethanol (v/v)) to the freshly harvested mycelium. The suspension was ground for 1 min using the VWR pellet mixer. 7 μL 20% (v/v) SDS and 26 μL 5 M KAc were added and the suspension was ground again for 1 min. The extraction samples were incubated for 10–60 minutes at 65°C followed by 10 minutes on ice. The samples were centrifuged for 10 minutes at 4°C at 16400 rpm using an Eppendorf Centrifuge 5417R, and the clear supernatant was transferred to a new tube. The centrifugation and transfer of supernatant was repeated. 128 μL ice-cold isopropanol and 12 μL 3 M NaAc were added to the samples, which were then incubated at −20°C for 10 minutes or longer. The samples were centrifuged for 5 minutes at 4°C at the maximal speed and the supernatant was discarded. The pellet was washed with 70% cold ethanol, air-dried and resuspended in MQ water to 100 μg mL^-1^.

To identify transformants that had integrated the *mttA* or *mfsA* gene, a PCR was carried out on the extracted DNA using primers specific for the *mttA* gene (Fw 5′CCC-GCA-AGT-ACA-GTA-AGA-ACG 3′ and Rv 5′CCT-GTA-CGG-AAC-CAG-ACT-CC 3′) and the *mfsA* gene (Fw 5′ TGA-TGG-GCT-CCT-TTA-ACT-GC 3′ and Rv 5′ GAT-AAG-ACC-GGC-GAT-AGT-GG 3′).

### DNA extraction and PCR of *cadA* + *mttA* + *mfsA A. niger* transformants

DNA extraction and PCR was carried out to identify the colonies that randomly integrated the genes of interest. Fresh mycelium was disrupted using Fastprep and 400 μL extraction buffer (100 mM TrisHCl pH 8.0, 5 mM EDTA, 1.2 M NaCl). DNA was extracted using phenol-chloroform, and the pellet was washed with 70% cold ethanol, air-dried and resuspended in 50 μL MQ water.

The identification of the transformants was carried out on the extracted DNA using PCR with Taq polymerase and the LS_*mttA*_for (5′- ATT-AAG-ACC-CGC-ATG-CAA-TC 3′) and LS_*mttA*_rev (5′- CTT-CTC-GTA-GAC-GGG-GAA-CA 3′) primers to check for the presence of the *mttA* gene. The LS_*mfsA*_for (5′- ACC-TTC-ACT-AGC-TGG-CGT-GT 3′) and LS_*mfsA*_rev (5′- GAC-ATC-CGT-GGG-ACT-GAA-CT 3′) primers were used to check for the presence of the *mfsA* gene.

### Growth experiments of transformants in shake flasks

All positively identified *cadA* + *mttA* and *cadA* + *mfsA* transformants were grown at 30°C and 200 rpm in 250 mL shake flasks containing 25 mL PM medium (1.2 g NaNO_3,_ 0.5 g KH_2_PO_4_, 0.2 g MgSO_4_^.^7 H_2_O, 0.5 g yeast extract and 40 μL Vishiniac per liter with 100 mM sorbitol as a carbon source [11]). Induction at t = 0 with 10 mM D-xylose was carried out 18 hours after inoculation. HPLC analysis was carried out on the samples after 30 hours.

Transformants containing the *cadA*, *mttA* and *mfsA* genes were grown in 1 L shake flasks containing 200 mL PM medium as described above. Samples were taken at 6, 30, 54 and 78 h after induction.

### Fermentation studies

The transformants containing the *cadA*, *mttA* and *mfsA* genes and the control strains, sCAD4, *cadA* + *mttA* 1.2 and *cadA* + *mfsA* 2.5, were inoculated (10^6^ spores/mL) in 1 L fermenters (Sartorius) containing 0.75 L of PM medium with 100 mM sorbitol. After 18 hours of growth at 30°C, the strains were induced by the addition of 50 mM xylose. The strains were further grown for 5 days at 30°C at a stirrer speed of 1000 rpm. The pH in the culture broth was not controlled. Samples were taken at 6, 30, 54 and 78 hours after induction.

### Dry weight measurement

To determine dry weight, 10 mL fermentation broth was sampled and filtered using a 5 micron nylon gauze. The biomass was washed with demineralized water and completely dried on pre-weighted aluminum trays in an oven at 120°C for 24 hours.

### HPLC analysis

High-pressure liquid chromatography (HPLC) was used to determine the extracellular concentrations of sorbitol, xylose, itaconic acid, citric acid, *cis*-aconitic acid, pyruvic acid, α-ketoglutaric acid, lactic acid, succinic acid, fumaric acid and oxalic acid in the samples. For organic acid measurements, a Shodex KC811 column was used and eluted with 0.01 N H_2_SO_4_ at a flow rate of 0.8 mL min^-1^ and sampling was carried out at 30°C for 25 min. Detection was carried out using both a refractive index detector (Spectra system RI-150, sample frequency 5.00032 Hz) and a UV–VIS detector (Spectrasystem UV1000, λ = 210 nm). 6 mM crotonate was used as an internal standard. The sugars were measured using a Dionex Carbopac MA-1 column.

### Determination of copy number

DNA was extracted using the method described in “DNA extraction and PCR of *cadA* + *mttA* + *mfsA* transformants”. The copy number of the *cadA* genes in the transformants expressing *cadA* from *A. terreus* or the synthetic codon-optimized *cadA* gene was determined in triplicate using a Rotor-Gene Q Cycler. The reaction mixture contained 8 μL 2× Absolute QPCR SYBR Green mix (Thermo Scientific), 100 nM forward and reverse primers and 2 μL 100 times diluted gDNA. Primers LS_qcadA_F (5′- GAGATCTTATGGCGGTTTCCTC - 3′) and LS_qcadA_R (5′- CAAGAGCTCGGGGTATCTCC - 3′) were used to determine the copy number of the *A. terreus cadA* gene and the primers LS_qcadAs_F (5′- ACTCCGAAGAGTTCGACCAG - 3′) and LS_ qcadAs_R (5′- ACCAGGTCCTCGATTTCCTT - 3′) were used to determine the copy number of the synthetic *cadA* gene. The *pkiA* gene, of which only one copy is present, was used as a reference gene using the primers LS_qpkiA_F (5′- GGTAACGACAGCGATTGGAT – 3′) and LS_qpkiA_R (5′- GGGCTCAAAGTGAATGTGGT - 3′). Water and SDS samples were used as controls. The qPCR cycling program was as follows: 15 min initial polymerase activation at 95°C followed by 40 cycles of 95°C for 15 sec, 59°C for 15 sec and 72°C for 30 sec. The calculations were carried out using the Pfaffl method [16].

## Competing interests

The authors declare that they have no competing interests.

## Authors’ contributions

LS, MV, ML, WB, TS, JC designed and performed the experimental work and participated in writing the manuscript. IM and AK collaborated in the coordination of the research and helped to draft the manuscript. LG designed the study and participated in writing of the manuscript. All authors read and approved the submission of the manuscript.
